# Reports on the Hospitals of the United Kingdom

**Published:** 1910-02-12

**Authors:** Henry Burdett


### REPORTS
ON THE
### Hospitals of the United Kingdom.

By SIR HENRY BURDETT, K.C.B., K.C.V.O.

### XXV.—THE ROYAL HALIFAX INFIRMARY.

This institution was founded in 1807, opened as a dispensary only in 1808, a few beds were added in 1815, and a new infirmary was built and opened in 1896. Between 1838 and 1891 the number of beds was raised from 70 to 100. The new hospital at present occupied was designed to accommodate about 300 patients, but when the new buildings were commenced in 1893 four pavilions only were erected, which contain 150 beds, of which 118 are constantly occupied. It was determined at that time to complete the whole of the administration building, so that the nurses could be there accommodated as well as the other female staff, in order to obviate the necessity of incurring the expense of erecting the nurses' home, which, though planned, has never yet been built.

The patients were first admitted to the new buildings in 1896. The site is open and attractive and the whole plan exhibits the maximum of care, ability, technical knowledge and foresight on the part of the architects, Messrs. Worthington and Ellgood, and their successors, Messrs. Thos. Worthington and Son, and does them infinite credit. This was practically the first pavilion hospital planned and erected where each ward unit was complete. Though opened 14 years ago, they fulfil every requirement except that one or two of the rooms might be larger, and that a clinical room has had to be added owing to the more recent developments in matters of treatment.

It was a great pleasure to inspect this hospital, the original plans having been submitted to us many years ago. As we passed through the buildings we were constantly reminded of the discussions and difficulties which were successfully overcome. This institution occupies an interesting position in the history of hospital construction, for it marked a new and great advance upon anything previously attempted.

### The General Condition of the Hospital.

Everywhere we found the most perfect order and smartness, save perhaps in the district nurses' room, a fact which rather indicates that district nurses are a little out of place inside a hospital unless they are kept strictly under the same discipline as other members of the staff. It was remarkable to note that every little detail had been carefully thought out, and that the discipline throughout the hospital was so good that every wardmaid and each member of the staff was keenly anxious to exhibit the maximum of efficiency in her work. It was even more important to find that they had succeeded in attaining this high standard. In the nurses' dining-room, for example, we watched the cutting of the bread and butter for the nurses' table, and were impressed by the circumstance that it was not cut until just before it was wanted and that it presented a tempting appearance which made it worthy of a queen's table. This hospital, which is under the able administration of Miss Huxley, who, be it noted, was trained at Halifax, must be an ideal place of residence for the staff, and, we should think, in many respects for the patients also. The laundry, stores, kitchens, wards, basements everywhere were in the most perfect order, and everything we tested was found to be of the best. Further, there was an absence of any extravagance, and the whole place seems to us to be smartly and economically administered. The system does great credit to its authors, whilst it bears remarkable testimony to the high efficiency of every member of the staff.

### A Few Points.

We have testified to the perfect cleanliness and order and the well-thought-out plan of the hospital. It may be well to add that the same foresight and thoughtfulness are exhibited in the whole of the plan which provides for future needs, so that as they arise the managers will be able to meet them with the minimum of cost and on the most efficient basis. The operation unit is complete and occupies a pavilion to itself. This was a new departure at the date of its erection. The terrazzo floor of the theatre had cracked, but the manufacturers had managed to restore it and to render it almost perfect, a fact to which we are able to testify for the first time in a long experience in work of this kind.

We thought that a little more furniture, especially an attractive and somewhat larger table, might be supplied to each of the wards. It is feared, however, that this common adjunct of a modern ward might be out of place at Halifax, for it might interfere with the free entrance and use of the patients' trolleys when they enter or leave a ward.

We noted that block tin has been used in lieu of slate and wood and porcelain for the side inclines to the ward scullery sinks. The result, in appearance, in wear and tear, and in freedom from all the usual objections is completely satisfactory. Block tin in this connection has been introduced into more than one of the northern hospitals, and we think it has established a reputation which should cause it to be accepted as the most suitable and best thing to use in this connection in every hospital in future.

Speaking generally, we found the Royal Halifax Infirmary a most attractive place and one which made us feel that it would be a pleasure again to enter actively the field of hospital administration and to take charge of this institution. We should like to complete the unbuilt portions of the original scheme. Descending to practical everyday considerations, we feel justified in declaring, as the result of a very careful inspection, that we regard this hospital under its present administrators as a delightful place to be associated with.

### XXVI.—THE BOLTON INFIRMARY.

This hospital was established in 1814, a new infirmary having been opened in 1885. It contains 138 beds, with an average occupancy of 128.

This hospital is noteworthy from the fact that its Secretary, Mr. James Briscoe, who has grown grey in its service, has been actively interested for many years in the work of raising contributions for the infirmary from the working men. In 1907 Bolton was ninth amongst the English towns in this respect, for its artisan population subscribed £16.86 per 1,000 of the population to the hospital. Mr. Briscoe is very deeply interested in the welfare of the hospital, and has much at heart the speedy enlargement of the balconies at the end of each ward, so as to enable patients to be treated there continuously. Having regard to the crowded condition of the wards and to the excessive number of beds which some of them contain, we hope Mr. Briscoe's scheme may be taken up warmly, and that the verandahs may be fixed and occupied by the summer of 1910. Forty beds might thus be added to the total, and, better still, the number of beds in the wards might be reduced to reasonable proportions by transferring some of them from the wards to the verandahs.

Another much needed improvement is the addition of a new casualty department with a small theatre and adequate recovery rooms. The expense entailed by the verandah and casualty additions, here recommended, need not be large, and having regard to their urgency, and to the fact that they would improve materially the hygienic condition of the wards, which at present are deficient in this respect, and at the same time add to the in-patient accommodation, we shall hope to hear that both additions have been put in hand. It would be a worthy recognition of the length of service of Mr. Briscoe for this reconstruction to be carried through by the date we have named.

### The General Condition.

Speaking generally, we should say that this infirmary suffers from the absence of adequate ventilation and strict administrative efficiency. With the exception of the newest wards—and even there, in converting the old out-patient department, a side ward has been taken in which has faulty ventilation—the atmosphere of the whole establishment is defective. We found the wards to contain too many beds, and to lack the free current and interchange of air which is essential to the well-being of patients confined to bed in a hospital. We are inclined to think that, apart from the excessive number of beds and the general overcrowding, the covering of the ward floors with linoleum is in a measure responsible for the relative inefficiency of the atmosphere. At any rate, it is perfectly certain that someone with a heart for the woes of little children should become interested in this infirmary, for the children's wards were some of the saddest we have ever inspected. One of these was formerly a ward kitchen, and it never ought to have been diverted from the purpose for which it was planned. It is unsuited in structure for use as a ward, and has no cross-ventilation of any kind. The worst ward was, however, a place which is called the Cot Ward. Here were several wee children shut up in a dull and unattractive little room with the door closed, from whence their cries could be indistinctly heard, and where, as far as we could observe, no nurse was present to look after and care for them. If those responsible are resolved to keep small children in such a place, a nurse ought to be constantly there to look after and reassure poor little mites like those we found occupying the cots.

The general condition of the wards follows, we presume, the local tradition which accepts the somewhat quaint lockers which we at first mistook for night stools. Generally an effort has been made recently to renew many of the fittings, and we found here an excellent porcelain bath, full size and well made, which had been supplied locally for the small cost of £7 10s. That is a notable fact, and if the whole of the work and the supplies are obtained on this economical principle great credit is due to the management. Another matter which should engage the attention of the medical staff and the committee is the number of operations which take place on a given day. It seems to us that they extend over too many hours, and that it would be better to have more days for operations with fewer operations on a given day. It must not be forgotten that the sister and nurses in charge of the theatre have to be on duty during the whole of the time, and so have the resident medical staff. We do not think there is any justification for keeping these staffs on duty for ten hours or even for eight hours a day in the operation theatre. If the operations during the ten hours are done by two or more surgeons they do not feel the pressure because their hours in the theatre are reasonable. The case of the residents and of the sister and nurses who have to be in the theatre the whole time is quite different, and ought to be thought of and provided for. This hospital did not present that smart appearance either in the wards or other departments which we look for nowadays in a modern up-to-date hospital. Our impression was that the whole of the Bolton Infirmary would benefit by the introduction of a little more outside influence and life. The whole establishment needs to be aroused and stirred up, and we direct the attention of the committee to this criticism with full confidence that they will consider it on its merits.

---

The **Tunbridge Wells General** and **Homœopathic Hospitals** will be reported on next week.

